# Ethical Principles and Practices in HIV Acquisition Research in Humanitarian Crises: A Cross-Sectional Study

**DOI:** 10.1017/jme.2026.10246

**Published:** 2026-03-10

**Authors:** Dina Garmroudi, Dini Harsono, Swarali Atre, Winnie Ho, Jacqueline Jin, Kate Nyhan, Hanna Peterson, J Lucian Davis, Kaveh Khoshnood

**Affiliations:** 1 Department of Chronic Disease Epidemiology, Yale School of Public Health, Yale University, New Haven, United States; 2 Center for Interdisciplinary Research on AIDS, Yale School of Public Health, Yale University, New Haven, United States; 3 Department of Internal Medicine, Yale School of Medicine, Yale University, New Haven, United States; 4 Department of Social & Behavioral Sciences, Yale School of Public Health, Yale University, United States; 5 Yale University, New Haven, United States; 6 Harvey Cushing/John Hay Whitney Medical Library, Yale University, New Haven, United States; 7 Department of Environmental Health Sciences, Yale School of Public Health, Yale University, New Haven, United States; 8 Department of Epidemiology of Microbial Diseases, Yale School of Public Health, Yale University, New Haven, United States; 9 Management Sciences for Health, Arlington, United States; 10 Department of Pulmonary, Critical Care and Sleep Medicine, Yale School of Medicine, Yale University, New Haven, United States

**Keywords:** ethics, research, HIV, humanitarian crises, cross-sectional studies

## Abstract

We conducted an exploratory cross-sectional analysis of ethical principles and practices in forty-one published research papers with empirical data on HIV prevalence, incidence, or risk factors in humanitarian settings. We identified ten key concepts pertinent to ethical principles and applications, and presented recommendations to inform future HIV prevention research.

## Introduction

Humanitarian crises caused by natural or human-made disasters affect more people today than ever. Recent estimates indicate that over 117 million people were affected by humanitarian crises worldwide due to conflict, human rights violations, natural disasters, climate change, and other events.^1^ Notable instances of conflict and violence include the Syrian Civil War^2^ and the Russia-Ukraine War,^3^ which have put 16.7 million and 14.6 million people in need of humanitarian assistance, respectively, due to mass displacement, widespread food insecurity, and damaged infrastructure. These humanitarian emergencies not only disrupt societal infrastructures but also amplify health vulnerabilities. The wars in Sudan^4^ and Gaza^5^ have severely strained the regions’ already fragile health systems and services, resulting in increasing mortality rates and creating new public health challenges. Natural disasters and extreme weather events, intensified by climate change, have also been strongly associated with negative health impacts, including increases in the frequency of infectious diseases, adverse respiratory disease outcomes, and mental health problems.^6^

Humanitarian crises may exacerbate a person’s risk of HIV infection through various mechanisms, including disrupted access to HIV prevention services, exposure to sexual violence, reliance on substance use as a coping strategy, and placing a low priority on HIV prevention and care compared to meeting basic needs such as food and shelter.^7^ In armed conflict and natural disaster settings where few sexual and reproductive health programs were available, adolescents and youth were at increased risk of sexual violence and exploitation, impacting their vulnerability to unwanted pregnancies and sexually transmitted infections (STI) including HIV.^8^ Among refugees and internally displaced individuals, food insecurity and reduced access to resources may drive participation in exchanging sex for money or non-monetary items as a means for survival.^9^ Natural disasters can also compound HIV transmission risk; for example, people with HIV (PWH) affected by flooding in Namibia were unable to access antiretroviral therapy (ART) and condoms due to a breakdown of infrastructure including lack of transport and damaged health facilities.^10^ Conducting empirical research on the impact of humanitarian crises on HIV acquisition is essential in order to understand the pathways of risks for affected individuals, and to inform HIV prevention strategies tailored to the emergency context.^11^ Such research may include assessments of how humanitarian crises affect stigmatized or criminalized behaviors (e.g., same-sex sexual behaviors, injecting drug use), traumatizing crisis-related experiences (e.g., gender-based violence, abduction or kidnapping), and potential consequences of receiving or disclosing an HIV-positive status including stigma and social exclusion among individuals with various mobility or migration status (e.g., refugee, internally displaced, asylum seeker).^12^ Investigators intending to gather data on these behaviors, health information, and experiences in humanitarian settings must address research-related ethical and safety issues to minimize harm and ensure the well-being of the involved individuals and communities.^13^

Given the rapidly evolving nature of humanitarian emergencies, it is critical to continuously review and adapt ethical guidelines to ensure both research integrity and protection of communities affected by humanitarian crises. Established codes of practice and guidelines — such as the Belmont Report^14^ and the International Ethical Guidelines for Health-Related Research Involving Human Subjects prepared by The Council for International Organizations of Medical Sciences (CIOMS)^15^ — provide critical guidance on the ethics of research involving human participants. These guidelines emphasize three core ethical principles: respect for persons (autonomy), beneficence (including non-maleficence), and distributive justice. These principles are implemented in research through informed consent, risk-benefit assessments, and equitable selection of participants. However, ethical guidelines specific to conducting health research in humanitarian crises remain limited. The CIOMS addressed this gap to some extent in its Guideline 20, which offers recommendations for research in disasters and disease outbreaks and acknowledges the complex balance between generating timely knowledge, maintaining public trust, and overcoming operational barriers during humanitarian crises.^16^ The World Health Organization (WHO) has also published recommendations to ensure that safety and ethical safeguards are in place before collecting data on sexual violence in emergency settings.^17^ Nevertheless, there is still a need to introduce research ethics frameworks that can provide practical advice and considerations for investigators and research ethics committees in developing and overseeing research protocols that engage humanitarian crisis-affected individuals, such as refugees, asylum seekers, and displaced individuals.^18^

Recent literature reviews have examined the ethical issues and considerations in conducting health research in humanitarian settings.^19^ To our knowledge, there is no rigorous synthesis of published data on the ethical principles and practices of HIV-related research in humanitarian settings. Thus, the current study aimed to systematically assess the ethical practices reported in HIV acquisition research within humanitarian settings — addressing a critical gap between ethical guidelines for research and practical application in crisis environments. Building on an existing scoping review project, we conducted an exploratory cross-sectional study of a representative sample of empirical research examining factors associated with HIV acquisition in humanitarian crises to map the extent of reporting ethical principles and practices in the published articles. Informed by the findings, we also sought to develop recommendations for ethical conduct and procedures for future HIV prevention research in humanitarian settings.

## Methods

### Study Design

The original scoping review aimed to identify and summarize evidence on modifiable and non-modifiable factors associated with HIV acquisition in the context of humanitarian crises.^20^ We followed the Joanna Briggs Institute (JBI) methodology for scoping reviews^21^ and the Preferred Reporting Items for Systematic Reviews and Meta-Analyses extension for Scoping Reviews (PRISMA-ScR) reporting guidelines.^22^ The present study was a cross-sectional analysis of ethical principles and practices reported in the empirical research articles identified in the original scoping review focused on factors associated with HIV acquisition among individuals affected by humanitarian crises. Factors associated with HIV acquisition were defined as a broad range of determinants that modify a person’s risk of HIV acquisition and/or contribute to HIV prevalence or incidence, including biological risk factors (e.g., non-HIV STI, chronic diseases), behavioral risk factors (e.g., alcohol or substance use, inconsistent condom use, exchanging sex for money), and structural variables (e.g., access to health facilities, employment status). Our team included public health professionals, an infectious disease epidemiologist, a pulmonary and critical care physician, and a research librarian who have taken part in conducting clinical and social-behavioral research and published peer-reviewed literature in the HIV/AIDS, humanitarian, and health sciences fields.

### Data Sources, Search Strategy, and Selection Criteria

We systematically searched bibliographic databases (i.e., MEDLINE, Embase, Global Health [all accessed via Ovid], and Scopus) between October 2021 and March 2022. We also sought out grey literature by conducting searches of humanitarian agencies and non-government organizations’ websites, the International AIDS Society’s abstract databases, and Google Scholar. The search strategy included terms for *participant* (e.g., refugee, asylum seeker, internally displaced person), *concept* (e.g., HIV infection/transmission, HIV incidence or prevalence, risk factor, sexual behavior), and *context* (e.g., earthquake, armed conflict, humanitarian). Inclusion criteria were articles that (1) involved individuals affected by humanitarian crises; (2) reported empirical data on HIV serostatus or risk factors of HIV acquisition as a primary or substantial focus; (3) were conducted in the context of humanitarian crises as defined by the United Nations, including *natural disasters* (e.g., floods, droughts) and *complex emergencies* associated with fragile states or areas of conflict (e.g., armed conflict, war);^23^ and (4) were published in English between January 1990 and March 2022. The full search strategy and resulting PRISMA-ScR flow diagram were previously published.^24^

### Data Screening and Extraction

We uploaded all identified citations to Covidence (Veritas Health Innovation, Melbourne, Australia) to manage the review process including deduplication, citation screening, full-text review, and data extraction. Two reviewers (Dini Harsono and Hanna Peterson) independently screened titles and abstracts and excluded articles that did not meet the inclusion criteria. Three reviewers (Swarali Atre, Dini Harsono, Hanna Peterson) conducted a full-text review of the remaining articles. Disagreements during screening and full-text assessment were discussed and resolved by consensus.

**Figure 1.**
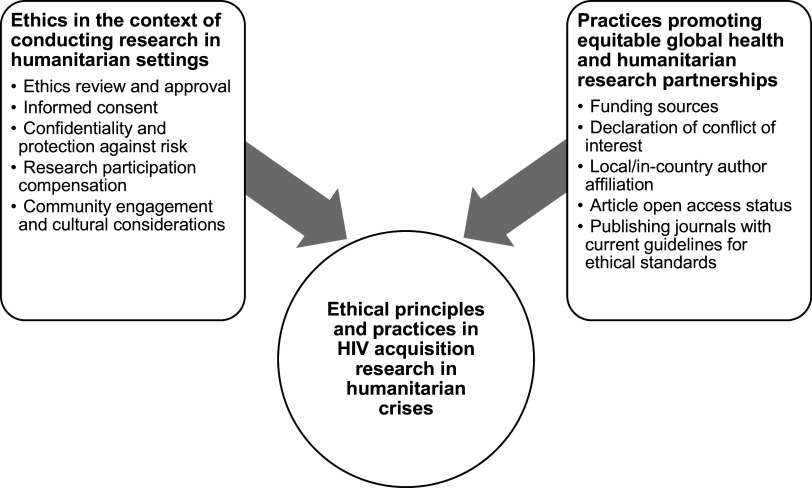
Selection of ethical principles and practices informed by humanitarian and global health literature.

Four reviewers (Swarali Atre, Dini Harsono, Hanna Peterson, Dina Garmroudi) conducted data extraction on study characteristics and key findings pertinent to the research question of the original scoping review. For the present cross-sectional study, two reviewers (Dini Harsono and Dina Garmroudi) extracted data pertinent to key concepts of ethical research conduct in the context of humanitarian settings. Our team developed a data extraction tool to capture these key concepts using a two-step process. First, informed by two highly cited review papers that provide robust discussion and important insights into ethical principles and issues in conducting research in humanitarian settings and engaging refugees and asylum seekers,^25^ we identified five key concepts, namely: ethics review and approval, informed consent, confidentiality and protection against risk, research participation compensation, and community engagement and cultural considerations. Second, guided by our team’s interest in fostering equitable global health research collaborations with a focus on humanitarian health, we reviewed literature on this topic to identify potential best practices. Humanitarian crises and forced displacement disproportionately affect populations residing in low- and middle-income countries.^26^ However, the majority of recipients of humanitarian-focused research and innovation funding are scientists or teams affiliated with institutions in high-income countries, highlighting inequalities in knowledge production that call for accessible resources for local researchers, groups, and communities impacted by humanitarian crises.^27^ Informed by literature centered on decolonization of global health and humanitarian research,^28^ we identified additional key concepts and extracted data on funding sources, declaration of conflict of interest, authorship affiliation, and the open access status of the published articles. Lastly, we also examined whether the journals in which the articles were published currently have guidelines that require authors to declare compliance with ethical standards — e.g., approval by Institutional Review Board or Research Ethics Committee (IRB/REC) or equivalent research ethics committee, informed consent, and disclosure of competing interests. In total, we reviewed ten key concepts pertinent to ethical principles and applications reported in the published articles (Figure 1).

### Data Synthesis

We summarized descriptive characteristics of included studies and developed a narrative summary of the ethical principles and practices reported in the studies. We distinguished between descriptive findings of ethical principles and practices explicitly reported in the studies that we present in the Results section, and normative analysis in the Discussion section in which we assess gaps in practice and make recommendations. We acknowledge that not all key concepts of ethical research would be reported in the articles, and that the absence of information does not necessarily equate to the absence of ethical principles and practices. If an article did not include the concept of interest, we classified it as “not mentioned” rather than “no” to avoid potential misrepresentation of the study. We addressed this as a limitation in the Discussion.

## Results

Forty-nine studies met the inclusion criteria of the original scoping review.^29^ Of these, eight were modeling studies of publicly available data or de-identified datasets where measures were taken to remove information that can be directly or indirectly used to determine the identity of an individual research participant. These studies were not subjected to an ethics review process; thus they were excluded from the current cross-sectional study (Supplementary Appendix, Figure S1). A total of 41 studies were included in the final analysis (Supplementary Appendix, References of included studies).

### Study Characteristics

Table 1 presents the characteristics of included studies. All studies (n=41) were published in peer-reviewed journals between 1994 and 2022. Study objectives reported by the authors included investigating the prevalence or incidence of HIV and risk behavior correlates of HIV infection (n=27, 65.9%), the prevalence or incidence and correlates of HIV and other infectious diseases (n=12, 29.3%), and the prevalence and correlates of mental health problems and HIV (n=2, 4.9%). Humanitarian settings of the studies included complex emergencies (n=35, 85.4%), natural disasters (n=2, 4.9%), complex emergencies and natural disasters (n=2, 4.9%), and not reported (n=2, 4.9%) (some percentages do not add up to 100% due to rounding). Most studies were conducted in low- and middle-income countries (LMIC) (n=36, 87.8%), primarily in sub-Saharan Africa (n=28, 68.3%). Study populations encompassed persons affected by conflict (n=19, 46.3%), persons affected by natural disasters (n=2, 4.9%), internally displaced persons (IDP) (n=2, 4.9%), military personnel (n=3, 7.3%), refugees (n=4, 9.8%), and police officers (n=1, 2.4%). Ten studies (24.4%) focused on more than one population group (e.g., IDP and refugees). Most studies enrolled both adults and children (under the age of 18) (n=27, 65.9%) and both female and male research participants (n=24, 58.5%). Most studies applied quantitative research methods (n=35, 85.4%) and employed cross-sectional designs (n=32, 78.1%).

**Table 1.** Characteristics of included studies (n=41) (some percentages do not add up to 100% due to rounding)

Study characteristic	N (%)
Humanitarian settings	
Complex emergencies	35 (85.4)
Natural disasters	2 (4.9)
Complex emergencies and natural disasters	2 (4.9)
Not mentioned	2 (4.9)
Geographical location^a^	
High-income countries	5 (12.2)
Low- and middle-income countries	36 (87.8)
Study population	
Persons affected by conflict	19 (46.3)
Persons affected by natural disasters	2 (4.9)
Multiple populations (e.g., IDP and refugees)	10 (24.4)
IDP	2 (4.9)
Military personnel	3 (7.3)
Refugees	4 (9.8)
Police officers	1 (2.4)
Age groups	
Adults (18 and older)	13 (31.7)
Adults (18 and older) and children (under the age of 18)	27 (65.9)
Not mentioned	1 (2.4)
Gender	
Female	13 (31.7)
Male	3 (7.3)
Female and male	24 (58.5)
Female, male, and transgender	1 (2.4)
Study methodology	
Quantitative	35 (85.4)
Qualitative	4 (9.8)
Mixed methods	2 (4.9)
Study design	
Cross-sectional	32 (78.1)
Prospective cohort	3 (7.3)
Case-control	2 (4.9)
Qualitative	4 (9.8)

Notes: *IDP* Internally displaced persons

a According to the World Bank classification. The geographical locations of the 41 studies were Afghanistan (1), Angola (1), Cameroon (1), Canada (2), Colombia (1), Democratic Republic of Congo (4), El Salvador (1), Ethiopia (1), Germany (1), Guinea-Bissau (1), Lebanon (1), Lesotho (1), Mozambique (1), Namibia (1), Nigeria (1), Pakistan (2), Rwanda (2), South Africa (1), South Sudan (1), Sudan (1), Thailand (1), Uganda (12), the United Kingdom (1), and the United States (1).

### Ethical Principles and Practices

**Figure 2.**
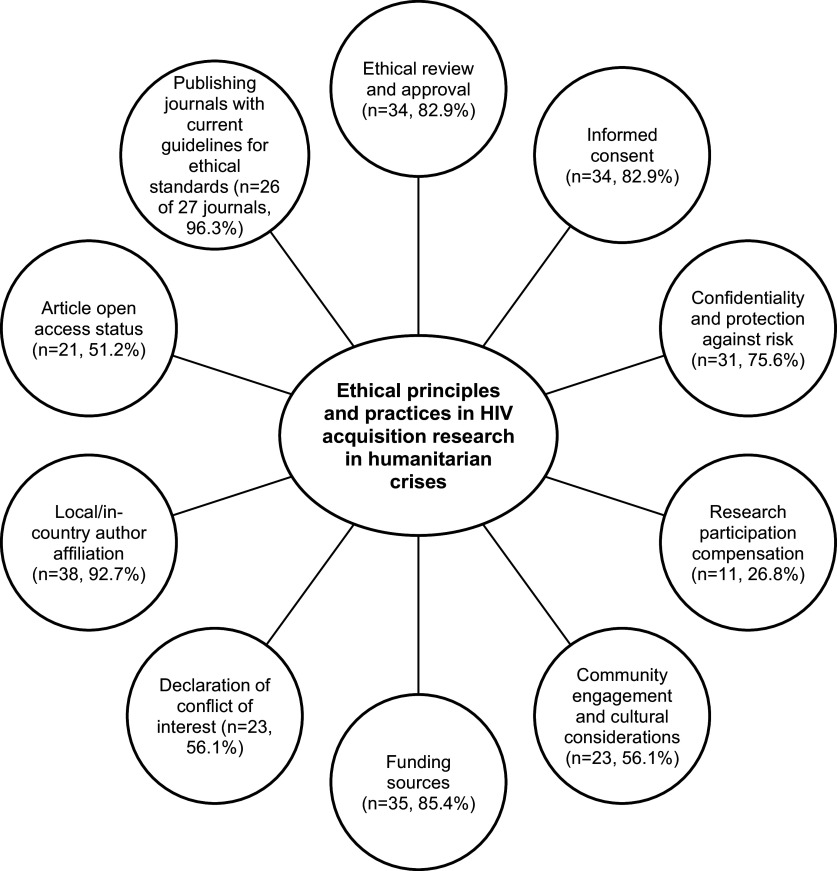
Frequency of key concepts concerning ethical principles and practices reported in the studies (n=41).

We identified ten key concepts pertinent to ethical principles and practices in the 41 studies (Figure 2). These key concepts were: ethics review and approval (n=34, 82.9%), informed consent (n=34, 82.9%), confidentiality and protection against risk (n=31, 75.6%), research participation compensation (n=11, 26.8%), community engagement and cultural considerations (n=23, 56.1%), funding source (n=35, 85.4%), declaration of conflict of interest (n=23, 56.1%), local/in-country authorship affiliation (n=38, 92.7%), article open access status (n=21, 51.2%), and publishing journal with current guidelines requiring compliance with ethical standards (n=26 of 27 journals, 96.3%). Table 2 provides a descriptive summary of ethical principles and practices reported in the included studies. A table charting the ethical principles and practices identified in each study can be found in the Supplementary Appendix (Table S1).

**Table 2.** Characteristics of ethical principles and practices of the included studies (n=41) (some percentages do not add up to 100% due to rounding)

Characteristic	Yes or not mentioned	Sub-characteristics	N (%)
Ethics review and approval type	Yes	IRB/REC	14 (34.1)
		IRB/REC and government agency (e.g., Ministry of Health, Office of the President)	13 (31.7)
		IRB/REC and non-government organization	3 (7.3)
		Government agency	3 (7.3)
		Government agency and non-government organization	1 (2.4)
	Not mentioned		7 (17.1)
Ethics review and approval location	Yes	Local/in-country	12 (29.3)
		Overseas/out-of-country	1 (2.4)
		Local and overseas	21 (51.2)
	Not mentioned		7 (17.1)
Informed consent and/or assent	Yes	Verbal	8 (19.5)
		Written	4 (9.8)
		Combination^a^	13 (31.7)
		Not specified	9 (22)
	Not mentioned		7 (17.1)
Confidentiality and protection against risk	Yes	Confidentiality	14 (34.1)
		Protection against risk	6 (14.6)
		Confidentiality and protection against risk	11 (26.8)
	Not mentioned		10 (24.4)
Research participant compensation	Yes	Monetary	6 (14.6)
		Non-monetary	2 (4.9)
		Not specified	3 (7.3)
	Not mentioned		30 (73.2)
Community engagement and cultural considerations	Yes	Community engagement	5 (12.2)
		Cultural considerations	3 (7.3)
		Community engagement and cultural considerations	15 (36.6)
	Not mentioned		18 (43.9)
Funding source	Yes	HIC - national governmental organization	20 (48.8)
		HIC - higher education institution	1 (2.4)
		HIC - multiple^b^	6 (14.6)
		Global - United Nations agency	1 (2.4)
		LMIC - health care organization	1 (2.4)
		LMIC - investigator self-funding	1 (2.4)
		LMIC - national governmental organization	4 (9.8)
		LMIC - non-government organization	1 (2.4)
	Not mentioned		6 (14.6)
Declaration of conflict of interests	Yes	No conflict	21 (51.2)
		Potential conflict	2 (4.9)
	Not mentioned		18 (43.9)
Authorship affiliation	LMIC-based studies (n=36)	First author with HIC affiliation	24 (58.5)
		First author with LMIC affiliation	9 (22)
		First author with HIC and LMIC affiliations	3 (7.3)
		Last author with HIC affiliation	28 (68.3)
		Last author with LMIC affiliation	6 (14.6)
		Last author with HIC and LMIC affiliations	2 (4.9)
	HIC-based studies (n=5)	First author with HIC affiliation	5 (12.2)
		Last author with HIC affiliation	5 (12.2)
Article open access status^c^	Yes	Gold	14 (34.1)
		Green	6 (14.6)
		Bronze	1 (2.4)
	No	Closed	20 (48.8)
Publishing journal with current guidelines requiring compliance with ethical standards (n=27)^d^	Yes (n=26 journals)	Ethics review and approval	26 (96.3)
		Informed consent	25 (92.6)
		Confidentiality and protection against risk	24 (88.9)
		Declaration of conflict of interests	26 (96.3)
		Funding sources	26 (96.3)
		Data sharing and/or availability	24 (88.9)
		Research participation categorization	6 (22.2)
	No (n=1 journal)	Health and safety procedures	2 (7.4)
			1 (3.7)

Notes: *IRB* Institutional Review Board, *REC* Research Ethics Committee, *HIC* High-income country, *LMIC* Low- and middle-income country

a Combination of informed consent methods included verbal, written, video, thumbprint, permission by head of household, and assent for individuals under the age of 18.

b Multiple funding indicated more than one funding source (e.g., national governmental organization and higher education institution; national government organization and non-government organization).

c Open access (OA) categories were defined as gold (i.e., published in a fully OA journal, freely available online for anyone to read on the publisher’s website, with an explicit open license), green (i.e., freely available in public repositories following authors depositing the accepted manuscript version of their paper), bronze (i.e., freely available on the journal publisher’s website without an explicit open license), and closed access (i.e., not freely available to the public).

d The 41 included studies were published in 27 individual journals. The descriptive counts of the ethical standard sub-characteristics were not mutually exclusive as a journal could require more than one ethical standard.

#### Ethics Review and Approval

Of the 41 articles, 34 (82.9%) reported having the study protocols reviewed and approved by an IRB/REC or equivalent research ethics committee and 7 (17.1%) did not report receiving an ethics approval. Of the 34 studies with ethics approvals, 14 received approvals from IRB/REC, 13 from both IRB/REC and government agencies (e.g., Ministry of Health), 3 from both IRB/REC and non-government organizations (NGO), 3 from a government agency, and 1 from both a government agency and NGO. Among the studies not reporting an ethics approval (n=7), six were conducted in LMIC (i.e., Democratic Republic of the Congo, El Salvador, Mozambique, Pakistan, Sudan, Thailand) and one was in a high-income country (United Kingdom).

Among the 30 studies conducted in LMIC with ethics approvals, the majority (n=21) received approval from both local/in-country and overseas IRB/REC. Other LMIC-based studies reported ethics approvals from local IRB/REC only (n=8) or foreign IRB/REC only (n=1). In addition to ethics approvals, some studies in Uganda also received a letter issued by the Office of the President and signed by the participating district authorities.^30^ Of 4 studies conducted in HIC with ethics approvals, 2 obtained approvals from hospital/health center IRB/REC, 1 from a university IRB/REC, and 1 from both hospital and university IRB/REC.

#### Informed Consent

Thirty-four (82.9%) of the 41 studies mentioned obtaining informed consent from research participants. Of the 7 studies that did not describe informed consent, two noted that eligible individuals “agreed to participate”^31^ or that participation was voluntary^32^ without specifying the consent process, two were retrospective database reviews,^33^ one utilized an anonymized publicly available database,^34^ and two did not provide an explanation.^35^ Among the 34 studies that reported informed consent, the methods were as follows: 8 studies had verbal consent, 4 had written consent, 13 had a combination of consent methods, and 9 studies did not specify the form of consent. An example of a multi-form research consent was the use of verbal and written instructions as well as seeking leadership support in a study involving male military personnel in Rwanda.^36^ Some studies also detailed strategies to accommodate research participants’ varied comprehension levels during the consent process. Five studies in Uganda reported employing a thumbprint option to indicate consent for those with limited literacy.^37^ Other studies employed local study team members who explained the study aims and consent forms to participants in the local languages.^38^

Of the 41 studies, 27 (65.9%) involved both adults and children as participants. Nine studies described additional steps to obtain consent from child participants. Eight studies obtained child participants’ informed assent in addition to parent/guardian’s written consent.^39^ In two Ugandan studies, youth aged 14–18 years could participate in the study if they were self-supporting (i.e., emancipated minors) and completed an “enhanced consent process,” although details of this process were not reported.^40^ Additionally, some studies in Uganda allowed emancipated individuals under 18 (i.e., married, pregnant, or had children) to independently provide consent without a parent/guardian’s permission, as defined by the Uganda National Council for Science and Technology.^41^

#### Confidentiality and Protection Against Risk

Regarding protection against risk, we categorized risks into research-specific risks (e.g., breach of confidentiality) and background or contextual risks (e.g., risk of HIV acquisition due to displacement). Thirty-one (75.6%) of the 41 studies described safeguarding efforts to minimize research participants’ possible research-related risks — including risk to confidentiality (n=14), potential harm during the course of the study (protection against risk) (n=6), and both potential risk to confidentiality and harm (n=11). Of the 25 studies with procedures to protect participants’ confidentiality and privacy, these measures include: conducting the data collection (e.g., interviews) in a private and secure setting or a location of participant’s choosing,^42^ using unique study identifiers to enhance data protection,^43^ implementing data anonymization,^44^ and utilizing secure research data storage and/or transfer.^45^ Additionally, in a study involving persons who inject drugs in Afghanistan, the researchers provided participants with a “laminated number ‘charm’ on a thick yarn necklace” as their identification, as well as a preferred or street nickname to enhance confidentiality and privacy.^46^

Of the 17 studies that described approaches to mitigating potential risks from participating in research, researchers also considered background or contextual risks. Some studies included a provision of voluntary HIV testing,^47^ HIV education material and/or post-test counseling,^48^ and a referral to a health facility or community organization for health care and/or psychosocial services.^49^ One study employed trained staff with nursing or trauma counseling background to conduct research interviews with women affected by conflict in Rwanda.^50^

#### Research Participation Compensation

Of the 41 studies included in the analysis, 11 (26.8%) offered some form of compensation to research participants. Of those 11 studies, 6 provided monetary compensation,^51^ 2 offered non-monetary items,^52^ and 3 did not describe the form of compensation.^53^ Non-monetary items included meals, transportation, and harm reduction supplies,^54^ as well as “resources and a bar of soap” as recommended by local partners.^55^ Of note, researchers who conducted a survey study on sexual practices and risk behaviors among Rwandan women affected by conflict commented that providing research incentives might have “influenced women to say they experienced sexual trauma during the genocide so they could be included in the study.”^56^ This was despite the researchers’ efforts to recruit potential participants through grassroots organizations supporting genocide survivors.^57^

#### Community Engagement and Cultural Considerations

Approximately half of the studies (n=23, 56.1%) incorporated community engagement and cultural considerations as part of their methodology — namely, measures to engage community members (n=5), strategies to ensure cultural sensitivity (n=3), and both aspects (n=15). Eight studies reported consulting with community leaders or members on research procedures.^58^ Some studies also reported conducting their research in collaboration with local stakeholders including healthcare facilities, NGOs, and individuals with lived experience.^59^ Lastly, 4 studies employed local guides or “community mobilizers” to facilitate recruitment.^60^

The primary approach to considering cultural perspectives reported in the studies was through language, by engaging local research staff members with fluency in local/regional languages^61^ or familiarity with local life^62^ to conduct participant recruitment, refine instruments, and administer surveys or interviews. Other approaches included translating study documents into local/regional language(s),^63^ engaging a local facilitator/translator in data analysis,^64^ and employing research instruments adapted to local settings.^65^ Studies also reported matching the gender of the interviewers and participants,^66^ organizing homogenous focus groups by ethnicity or HIV status,^67^ and employing research staff trained in addressing sensitive topics such as war-related trauma.^68^

#### Funding Source

Thirty-five (85.4%) of 41 studies declared a funding source. Among 31 studies conducted in LMIC reporting funding sources, most (n=23) received funding from organizations located in high-income countries (HIC) such as the United States National Institutes of Health or the Canadian Institutes of Health Research. The other 8 LMIC-based studies stated receiving funding from local governmental entity or NGO (n=7) and a United Nations agency (n=1). Four studies conducted in HIC that received funding mentioned national governmental organizations (n=2) and a mix of funding sources (n=2).

#### Declaration of Conflict of Interest

Of the 41 studies, 23 (56.1%) included information on whether the authors had any conflict of interest and 18 did not state such information. Among the 23 studies with conflict of interest statements, 21 declared no conflict and 2 acknowledged potential competing interests. Two of the 8 authors in one study were affiliated with a national military agency.^69^ In another study with 11 authors, one author was affiliated with a national government organization and two other authors reported receiving funding from a pharmaceutical company that was separate from the published study.^70^

#### Authorship Affiliation

We examined author affiliations of the research articles, specifically the first and last authors’ affiliations with institutions located in HIC and LMIC. Regarding first authorship, most (n=29, 70.1%) of the 41 articles were published by HIC-affiliated first authors, with the most frequent HIC institutions in the United States (n=12) and Canada (n=9). Other studies had a first author with LMIC affiliation (n=9, 22%) and a first author with dual affiliation with both LMIC and HIC institutions (n=3, 7.3%). A similar trend was observed regarding last or senior authorship, with the majority of articles (n=33, 80.5%) published by last authors affiliated with HIC institutions, notably in the United States (n=14) and Canada (n=11). Six (14.6%) of the 41 articles had LMIC-based last authors, and two (4.9%) articles had last authors with both LMIC and HIC affiliations.

A further examination on author affiliations according to research location found that of the 36 studies conducted in LMIC, 33 (91.7%) studies had at least one author with LMIC affiliation, an indication of collaborative research partnerships between HIC- and LMIC-based institutions. An analysis of first and last authorship of these LMIC studies (n=36) saw that the majority were published by HIC-based first authors (n=24, 66.7%) and HIC-based last authors (n=28, 77.8%). Lastly, of the 5 studies conducted in HIC, all were published by first authors with HIC affiliations.

#### Article Open Access Status

We examined whether the included research articles were available as open access (OA) publications, defined as being accessible to the public electronically at no cost, as of December 2024. Utilizing OpenAlex (https://openalex.org/) to retrieve the OA status of the 41 publications — further verified by conducting manual searches of each article on Google — we found that 21 (51.2%) articles were freely available for access through different tiers of OA, and 20 articles were not freely available to the public (i.e., closed access). Among the 21 articles with OA, 14 were gold OA (i.e., published in a fully OA journal, freely available online for anyone to read on the publisher’s website, with an explicit open license), 6 were green OA (i.e., freely available in public repositories following authors depositing the accepted manuscript version of their paper), and 1 was bronze OA (i.e., freely available on the journal publisher’s website without an explicit open license). None of the included articles fell in the diamond OA category (i.e., freely available in journals that do not charge fees to either authors or readers) or hybrid OA category (i.e., available on the journal publisher’s website through subscription, with an option for authors to pay a fee to make their article OA). Of the 36 studies conducted in LMIC, half (n=18, 50%) were available with OA (12 gold, 5 green, 1 bronze). Of the 5 HIC-based studies, 3 were available with OA (2 gold, 1 green).

#### Journal Guidelines Requiring Compliance with Ethical Standards

Of 27 journals in which the 41 studies were published, all but one currently stated in the submission guidelines a requirement to comply with ethical standards (as of December 2024). The six ethical standards most commonly required to report in the 27 journals were ethics review and approval (n=26, 96.3%), informed consent (n=25, 92.6%), confidentiality and protection against risk (n=24, 88.9%), conflict of interest (n=26, 96.3%), funding sources (n=26, 96.3%), and data sharing and/or availability (n=24, 88.9%). Additionally, 6 journals (22%) required a description of how authors categorized research participants according to race/ethnicity, gender, sexual orientation, and other social and cultural variables. Two journals (7.4%) also required authors to confirm that health and safety procedures were fulfilled during the study. Although one journal (3.7%) did not specify a requirement to report ethical standards in its submission guidelines, we found that the published study included in our analysis reported aspects related to ethics approval, informed consent, confidentiality and protection against risk, and funding source.^71^

## Discussion

As part of a larger scoping review project, we conducted an exploratory cross-sectional analysis to map the extent of reporting ethical principles and practices in published research papers with empirical data on HIV prevalence, incidence, or risk factors among humanitarian crisis-affected populations. We extracted and summarized data related to ten key concepts of ethical principles and practices, including ethics review and approval, informed consent, confidentiality and protection against risk, compensating research participants, community engagement and cultural consideration, funding sources, declaration of conflict of interest, authorship affiliation, article open access status, and journal guidelines for ethical standards. To our knowledge, this is the first study focusing on the ethics of HIV acquisition research in humanitarian settings.

The diversity in ethical practices observed across studies underscores both innovative approaches and significant gaps that warrant further discussion. Most HIV acquisition studies documented obtaining ethical approvals from an IRB/REC located where the research was conducted, predominantly in LMIC. Of the 30 LMIC-based studies with ethics approvals, the majority (70%) received ethics approvals from both local and overseas IRB/REC. For an LMIC-based study with a research team comprised of both LMIC- and HIC-affiliated investigators, obtaining ethical approvals from both jurisdictions can help ensure that the proposed research activities align with the values and cultural norms of local communities.^72^ While the dual IRB approval can potentially introduce procedural delays that must be managed carefully, obtaining such approval is an important step to help minimize risks to participants, foster ethical research practices, and promote equitable collaboration.^73^

Our analysis found that while most studies specified their informed consent process, including strategies to accommodate study participants’ comprehension levels, some studies did not describe how informed consent was obtained. Ensuring participants understand the purpose of the study and their potential involvement is particularly crucial when engaging vulnerable individuals such as those affected by humanitarian crises^74^ and addressing sensitive topics such as HIV risk behaviors^75^ and mental health diagnoses.^76^ Informed consent process in humanitarian settings can also be complicated by language barriers and power imbalances. Tailored strategies such as engaging local facilitators and employing enhanced consent procedures are essential to ensure true comprehension and voluntariness. Regarding research involving children as participants, only 9 out of 27 studies described additional consent processes where parental consent was obtained prior to seeking the child’s informed assent. Obtaining parental consent to engage children in research can be challenging in humanitarian settings as the affected community may be at risk to coercion and inadequate consent processes;^77^ thus, we recommend that IRB/REC specify what youth-specific informed consent processes should entail in this situation and provide guidelines that can enable researchers to effectively adopt and implement these practices. Examples of such practices may include implementing informed consent as an ongoing process that is responsive to rapid changes commonly occurring in humanitarian settings,^78^ tailoring consent strategies according to the children and youth participants’ age and developmental stages,^79^ and employing study team members who are trained in detecting and responding to childhood distress (e.g., social worker, psychologist).^80^

We found that most studies had implemented suitable practices to protect research participants’ confidentiality — such as conducting interviews in a private and secure setting, and enhancing data protection by using unique study identifiers, data anonymization, and secure research-data storage. Researchers also implemented strategies to minimize possible harms to research participants by offering voluntary HIV testing and counseling and a referral to counseling or psychological support. In studies examining sensitive topics such as exposure to sexual violence or HIV risk behaviors among children and youth participants, HIV researchers should also be aware of local laws pertaining to the legal age of consent to research, and whether waivers of parental permission may be considered (e.g., when the research poses minimal risk) to protect the children’s rights to privacy and confidentiality as determined by the local or national regulatory authorities and IRB/REC.^81^

We found that less than one-third of the studies (26.8%) reported compensating participants for their time and research-related inconveniences. Individuals affected by humanitarian crises may be vulnerable due to powerlessness and lack of resources to make voluntary choices:^82^ researchers conducting a survey among women experiencing genocide in Rwanda pointed out their concern about providing compensation that might have influenced some participants to assert sexual trauma experiences in order to participate in the study.^83^ Among the few studies that provided compensation, only one study described seeking input from local partners to determine the suitable form of compensation.^84^ While compensation for research participants is considered appropriate under many circumstances in order to acknowledge participants’ time, its application must be carefully calibrated to avoid undue influence — especially in contexts where economic vulnerabilities may affect decision-making. We recommend that compensation must balance fairness, respect, and contextual sensitivity — recognizing both the possibility of coercion in research participation, and the ethical imperative to acknowledge participants’ burdens of participation (e.g., time, inconvenience) and contributions.^85^ We also recommend researchers consult with local representatives to determine compensation rates that are in line with local norms and practices, and carry out adequate informed consent to prevent unduly influencing economically vulnerable individuals to participate in research.^86^ Specific to research in humanitarian settings, potential participants should be informed that their research participation is voluntary and that the humanitarian aid or resources they receive would not be affected by whether or not they participate in research. Collaborating with local representatives and organizations to convey the differences between humanitarian assistance and research participation with sensitivity to local customs can optimize accurate communication and minimize misinterpretation.

Around half of the studies (56.1%) described approaches to engaging community and considering local cultures as part of their methodology, including consulting with community leaders to ensure respect for sociocultural context and collaborating with local organizations and guides. The depth and consistency of these approaches varied considerably, suggesting the need for a more standardized engagement protocol. A supplementary research ethics framework could be developed to offer guidance on engaging individuals affected by humanitarian crises in health research, including HIV-related research. Such guidance may include the ten key concepts pertinent to ethical principles and applications examined in the current study, and practices that promote the needs and rights of communities affected by humanitarian crises — for example, determining the social value of research that considers the feasibility of conducting the proposed research in non-disaster context and potential application of findings to inform future disaster responses,^87^ having refugees as members in the IRB/REC to review research protocols and consider risks and benefits of research participation,^88^ and establishing a community advisory board to inform study design and methodology from project conception through dissemination.^89^ Further, drawing from a systematic review of ethical guidelines for research in disaster situations by Mezinska and colleagues,^90^ we recommend researchers consider the vulnerability of research participants that may occur in the humanitarian settings — due to political status, human rights violations, physical and mental health consequences, and poverty and disempowerment — to ensure that appropriate research participation protection plans are put in place and tailored to the specific context of humanitarian crises.

Ensuring transparency is crucial for maintaining trust in the scientific process, including when disseminating research findings to research participants, community partners, collaborating investigators, providers, funding agencies, and the broader public. We assessed different aspects related to enhancing transparency and trust in the scientific literature reported in the reviewed studies concerning funding sources: declaration of conflict of interest, authorship affiliation, article open access status, and journal requirements to ethical standards compliance. Most studies (85.4%) stated a funding source(s), with the majority of LMIC-based studies (23 out of 31 studies) receiving funding from organizations located in HIC. Half (56.1%) of the published research articles had conflict of interest statements. Despite a consensus calling for disclosure to mitigate the risk of bias in the study design, research conduct, and publishing results, conflict of interest remains underreported.^91^ Stricter journal enforcement and clearer guidelines could improve the transparency regarding funding and potential biases. Regarding authorship affiliation, we found that the majority (91.7%) of LMIC-based HIV acquisition studies involved collaborations between HIC and LMIC investigators. The first and last authorships of these LMIC-based studies (n=36) were dominated by HIC-affiliated authors, 66.7% and 77.8%, respectively. The predominance of HIC-affiliated first and last authors in LMIC-based studies raises concerns about equitable research partnerships. Efforts to ensure that local expertise is fully recognized and to enhance authorship equity in global health publications may include facilitating shared authorship — for example, having co-first or co-last authorship or LMIC author serving as first author under the mentorship of HIC last author or vice versa.^92^ Further, disseminating research findings in ways that are meaningful and accessible to the affected communities is especially critical in humanitarian settings, where transparency and trust are paramount. With only around half (51.2%) of the studies published as open access articles and the prohibitive publication fees often identified as a barrier to publishing in prestigious journals,^93^ authors could consider ways to ensure public access to their published research — for example, by obtaining waivers for article processing charges or publishing in the diamond open access journals. Authors should also utilize other dissemination strategies to promote the uptake of research-generated evidence in practice and policy among key stakeholders — such as creating infographics, issue briefs, and press releases, or having one-on-one meetings with a policymaker or elected official.^94^ Of the 41 HIV acquisition studies, 2 reported changes made to the HIV prevention programs in the communities where the studies were conducted. In a study involving military personnel in Sudan that found high rates of probable alcohol dependence, the military agency enhanced their HIV prevention program by incorporating an alcohol risk reduction component.^95^ Following a prevalence study of HIV and syphilis in a refugee camp in Thailand, the on-site HIV prevention of mother-to-child transmission (PMTCT) program implemented opt-out instead of opt-in HIV testing, and provided routine syphilis testing and education on sexually transmitted infections.^96^ Lastly, with varying ethical standards described in the journal guidelines in which the studies were published, we suggest following the International Committee of Medical Journal Editors (ICMJE)’s recommendations for manuscript publication that promote responsible and transparent approaches to authorship, publication, and editorial decisions.^97^

The current study has several important strengths. First, our efforts to identify relevant studies addressing HIV acquisition in humanitarian crises were conducted in a systematic and reproducible manner and included both peer-reviewed journals and grey literature. Second, all review processes — including record screening, full-text review, data extraction, and summary and presentation of findings — were conducted collaboratively by multidisciplinary study team members with expertise in infectious disease epidemiology, research ethics, public health, and evidence synthesis. Third, our identification of key concepts of ethical research conduct were informed by published reviews on research ethics in humanitarian settings and refugee health literature as well as through team discussion and consensus. The current study also has some limitations. First, we did not include non-English-language articles due to time and human resource constraints. Second, some ethics considerations may not be reported in the published articles given the individual journals’ differing requirements in their submission guidelines (as of December 2024 when we reviewed the guidelines). Thus, given the exploratory nature of the present analysis, our findings were limited to the ethical principles and practices as they were reported by authors within the published articles; therefore, the results may underestimate the actual adherence to ethical standards and restrict our ability to make definitive normative claims. Lastly, while members of our team represent interdisciplinary expertise and experiences, our team did not include an ethicist whose perspectives could have provided additional insights into ethical principles and practices relevant to the current study.

## Conclusion

This study highlights significant variability in the reporting of ethical principles and practices in HIV acquisition research conducted within humanitarian settings. To bridge this gap, we recommend that researchers adopt standardized protocols for informed consent, confidentiality and protection against risk, conflict of interest disclosures, and community engagement and cultural considerations in order to promote the needs and rights of communities affected by humanitarian crises. Such measures will not only safeguard these vulnerable populations but also improve the overall integrity of research findings. Moreover, academic journals should consider requiring compliance with ethical standards in publications more rigorously. Future efforts should also focus on fostering a robust framework for ethical conduct of health research in humanitarian settings that offer practical advice and considerations for researchers and IRB/REC in developing and overseeing research protocols that engage crisis-affected populations.

## Supporting information

10.1017/jme.2026.10246.sm001 Garmroudi et al. supplementary material Garmroudi et al. supplementary material

## Supplementary material

The supplementary material for this article can be found at http://doi.org/10.1017/jme.2026.10246.
