# Supplementary material for: Ethical Principles and Practices in HIV Acquisition Research in Humanitarian Crises: A Cross-Sectional Study
Source: J Law Med Ethics. 2026 Mar 10:1–13. Online ahead of print. doi: 10.1017/jme.2026.10246 (PMC13040380; doi:10.1017/jme.2026.10246)
Supplement: Garmroudi et al. supplementary material [file S1073110526102460sup001.docx]

**Supplementary Appendix (Figure S1). Flow of cross-sectional analysis of ethical principles and practices reported in the studies.**

**Supplementary Appendix. References of included studies**

1. Adedimeji, A. A., D. R. Hoover, Q. Shi, T. Gard, E. Mutimura, Jd Sinayobye, M. H. Cohen, and K. Anastos. "Sexual Behavior and Risk Practices of HIV Positive and HIV Negative Rwandan Women." AIDS Behav 19, no. 7 (Jul 2015): 1366-78.
2. Alberer, M., S. Malinowski, L. Sanftenberg, and J. Schelling. "Notifiable Infectious Diseases in Refugees and Asylum Seekers: Experience from a Major Reception Center in Munich, Germany." Infection 46, no. 3 (Jun 2018): 375-83.
3. Aliyu, G. G., S. H. Aliyu, A. Ehoche, D. Dongarwar, R. A. Yusuf, M. H. Aliyu, and H. M. Salihu. "The Burden of HIV, Hepatitis B and Hepatitis C by Armed Conflict Setting: The Nigeria Aids Indicator and Impact Survey, 2018." Ann Glob Health 87, no. 1 (Jun 25 2021): 53.
4. Anthonj, C., O. T. Nkongolo, P. Schmitz, J. N. Hango, and T. Kistemann. "The Impact of Flooding on People Living with HIV: A Case Study from the Ohangwena Region, Namibia." Glob Health Action 8 (2015): 26441.
5. Beckwith, C. G., A. K. DeLong, S. F. Desjardins, F. Gillani, L. Bazerman, J. A. Mitty, H. Ross, and S. Cu-Uvin. "HIV Infection in Refugees: A Case-Control Analysis of Refugees in Rhode Island." Int J Infect Dis 13, no. 2 (Mar 2009): 186-92.
6. Cossa, H. A., S. Gloyd, R. G. Vaz, E. Folgosa, E. Simbine, M. Diniz, and J. K. Kreiss. "Syphilis and HIV Infection among Displaced Pregnant Women in Rural Mozambique." Int J STD AIDS 5, no. 2 (Mar-Apr 1994): 117-23.
7. Courtney, L. P., N. Goco, J. Woja, T. Farris, C. Cummiskey, E. Smith, L. Makuach, and H. M. Chun. "HIV Prevalence and Behavioral Risk Factors in the Sudan People's Liberation Army: Data from South Sudan." PLoS One 12, no. 11 (2017): e0187689.
8. Crawshaw, A. F., M. Pareek, J. Were, S. Schillinger, O. Gorbacheva, K. P. Wickramage, S. Mandal, et al. "Infectious Disease Testing of UK-Bound Refugees: A Population-Based, Cross-Sectional Study." BMC Med 16, no. 1 (Aug 28 2018): 143.
9. Fabiani, M., B. Nattabi, C. Pierotti, F. Ciantia, A. A. Opio, J. Musinguzi, E. O. Ayella, and S. Declich. "HIV-1 Prevalence and Factors Associated with Infection in the Conflict-Affected Region of North Uganda." Confl Health 1 (Mar 1 2007): 3.
10. Goldenberg, S. M., G. Muzaaya, M. Akello, M. Braschel, J. Birungi, and K. Shannon. "High Burden of Previously Undiagnosed HIV Infections and Gaps in HIV Care Cascade for Conflict-Affected Female Sex Workers in Northern Uganda." Int J STD AIDS 30, no. 3 (Mar 2019): 275-83.
11. Goldenberg, S. M., G. Muzaaya, M. Akello, P. Nguyen, J. Birungi, and K. Shannon. "War-Related Abduction and History of Incarceration Linked to High Burden of HIV among Female Sex Workers in Conflict-Affected Northern Uganda." J Acquir Immune Defic Syndr 73, no. 1 (Sep 1 2016): 109-16.
12. Haffejee, F., and K. Maksudi. "Understanding the Risk Factors for HIV Acquisition among Refugee Women in South Africa." AIDS Care 32, no. 1 (Jan 2020): 37-42.
13. Harbertson, J., M. Grillo, E. Zimulinda, C. Murego, T. Cronan, S. May, S. Brodine, et al. "Prevalence of PTSD and Depression, and Associated Sexual Risk Factors, among Male Rwanda Defense Forces Military Personnel." Trop Med Int Health 18, no. 8 (Aug 2013): 925-33.
14. Heimer, R., R. Barbour, D. Khouri, F. W. Crawford, F. Shebl, E. Aaraj, and K. Khoshnood. "HIV Risk, Prevalence, and Access to Care among Men Who Have Sex with Men in Lebanon." AIDS Res Hum Retroviruses 33, no. 11 (Nov 2017): 1149-54.
15. Holt, B. Y., P. Effler, W. Brady, J. Friday, E. Belay, K. Parker, and M. Toole. "Planning Sti/HIV Prevention among Refugees and Mobile Populations: Situation Assessment of Sudanese Refugees." Disasters 27, no. 1 (Mar 2003): 1-15.
16. Kaiser, R., T. Kedamo, J. Lane, G. Kessia, R. Downing, T. Handzel, E. Marum, et al. "HIV, Syphilis, Herpes Simplex Virus 2, and Behavioral Surveillance among Conflict-Affected Populations in Yei and Rumbek, Southern Sudan." AIDS 20, no. 6 (Apr 4 2006): 942-4.
17. Katamba, A., M. D. Ogwang, D. S. Zamar, H. Muyinda, A. Oneka, S. Atim, K. Jongbloed, et al. "Cango Lyec (Healing the Elephant): HIV Incidence in Post-Conflict Northern Uganda." eClinicalMedicine 23 (Jun 2020): 100408.
18. Khanani, M. R., A. S. Ansari, S. Khan, M. Somani, S. U. Kazmi, and S. H. Ali. "Concentrated Epidemics of HIV, HCV, and HBV among Afghan Refugees." J Infect 61, no. 5 (Nov 2010): 434-7.
19. Kim, A. A., F. Malele, R. Kaiser, N. Mama, T. Kinkela, J. C. Mantshumba, M. Hynes, et al. "HIV Infection among Internally Displaced Women and Women Residing in River Populations Along the Congo River, Democratic Republic of Congo." AIDS Behav 13, no. 5 (Oct 2009): 914-20.
20. Kowo, M. P., C. N. Frungwa, S. R. S. Njonnou, F. A. Andoulo, A. W. N. Ndam, L. D. Yemeli, H. C. N. Djeunga, C. Kouanfack, and J. Kamgno. "Epidemiologic Patterns of HIV, Hepatitis B and C Virus Infections among Refugees of the Mbile Camp in the East Region of Cameroon (Hepatitis and HIV among Refugees)." J Gastroenterol Hepatol Res 10, no. 3 (2021): 3524-30.
21. Low, A. J., K. Frederix, S. McCracken, S. Manyau, E. Gummerson, E. Radin, S. Davia, et al. "Association between Severe Drought and HIV Prevention and Care Behaviors in Lesotho: A Population-Based Survey 2016-2017." PLoS Med 16, no. 1 (Jan 2019): e1002727.
22. Luo, J., D. S. Zamar, M. D. Ogwang, H. Muyinda, S. S. Malamba, A. Katamba, K. Jongbloed, et al. "Cango Lyec (Healing the Elephant): Probable Post-Traumatic Stress Disorder (PTSD) and Depression in Northern Uganda Five Years after a Violent Conflict." J Migr Health 6 (2022): 100125.
23. Malamba, S. S., H. Muyinda, P. M. Spittal, J. P. Ekwaru, N. Kiwanuka, M. D. Ogwang, P. Odong, et al. ""The Cango Lyec Project - Healing the Elephant": HIV Related Vulnerabilities of Post-Conflict Affected Populations Aged 13-49 Years Living in Three Mid-Northern Uganda Districts." BMC Infect Dis 16, no. 1 (Nov 21 2016): 690.
24. Mansson, F., A. Biague, Z. J. da Silva, F. Dias, L. A. Nilsson, S. Andersson, E. M. Fenyo, and H. Norrgren. "Prevalence and Incidence of HIV-1 and HIV-2 before, During and after a Civil War in an Occupational Cohort in Guinea-Bissau, West Africa." AIDS 23, no. 12 (Jul 31 2009): 1575-82.
25. Mootz, J. J., O. A. Odejimi, A. Bhattacharya, B. Kann, J. Ettelbrick, M. Mello, M. L. Wainberg, and K. Khoshnood. "Transactional Sex Work and HIV among Women in Conflict-Affected Northeastern Uganda: A Population-Based Study." Confl Health 16, no. 1 (Feb 25 2022): 8.
26. Mulanga-Kabeya, C., N. Nzilambi, B. Edidi, M. Minlangu, T. Tshimpaka, L. Kambembo, L. Atibu, et al. "Evidence of Stable HIV Seroprevalences in Selected Populations in the Democratic Republic of the Congo." AIDS 12, no. 8 (May 28 1998): 905-10.
27. Mulanga, C., S. E. Bazepeo, J. K. Mwamba, C. Butel, J. W. Tshimpaka, M. Kashi, F. Lepira, et al. "Political and Socioeconomic Instability: How Does It Affect HIV? A Case Study in the Democratic Republic of Congo." AIDS 18, no. 5 (Mar 26 2004): 832-4.
28. Najib, Raheela, Shahina Mumtaz, Jawad Ahmed, Hube Muhammad, Nade Ahmed, and Muhammad Salman Haider Qureshi. "Frequency of Hepatitis B, Hepatitis C and Human Immunodeficiency Viruses in Internally Displaced Persons of South Waziristan, Pakistan." Journal of Postgraduate Medical Institute 31, no. 1 (2017): 82-87.
29. O'Laughlin, K. N., D. J. Rabideau, J. Kasozi, R. A. Parker, N. D. Bustamante, Z. M. Faustin, K. E. Greenwald, R. P. Walensky, and I. V. Bassett. "Predictors of HIV Infection: A Prospective HIV Screening Study in a Ugandan Refugee Settlement." BMC Infect Dis 16, no. 1 (Nov 23 2016): 695.
30. Patel 2014a. Patel, S., M. T. Schechter, N. K. Sewankambo, S. Atim, N. Kiwanuka, and P. M. Spittal. "Lost in Transition: HIV Prevalence and Correlates of Infection among Young People Living in Post-Emergency Phase Transit Camps in Gulu District, Northern Uganda." PLoS One 9, no. 2 (2014): e89786.
31. Patel 2014b. Patel, S., M. T. Schechter, N. K. Sewankambo, S. Atim, S. Lakor, N. Kiwanuka, and P. M. Spittal. "War and HIV: Sex and Gender Differences in Risk Behaviour among Young Men and Women in Post-Conflict Gulu District, Northern Uganda." Glob Public Health 9, no. 3 (2014): 325-41.
32. Patel, S., M. T. Schechter, N. K. Sewankambo, S. Atim, C. Oboya, N. Kiwanuka, and P. M. Spittal. "Comparison of HIV-Related Vulnerabilities between Former Child Soldiers and Children Never Abducted by the LRA in Northern Uganda." Confl Health 7, no. 1 (Aug 7 2013): 17.
33. Plewes, K., T. Lee, L. Kajeechewa, M. M. Thwin, S. J. Lee, V. I. Carrara, F. Nosten, and R. McGready. "Low Seroprevalence of HIV and Syphilis in Pregnant Women in Refugee Camps on the Thai-Burma Border." Int J STD AIDS 19, no. 12 (Dec 2008): 833-7.
34. Spittal, P. M., S. S. Malamba, M. D. Ogwang, S. Musisi, J. P. Ekwaru, N. K. Sewankambo, M. E. Pearce, et al. "Cango Lyec (Healing the Elephant): Gender Differences in HIV Infection in Post-Conflict Northern Uganda." J Acquir Immune Defic Syndr 78, no. 3 (Jul 1 2018): 257-68.
35. Strand, R. T., L. Fernandes Dias, S. Bergström, and S. Andersson. "Unexpected Low Prevalence of HIV among Fertile Women in Luanda, Angola. Does War Prevent the Spread of HIV?" Int J STD AIDS 18, no. 7 (Jul 2007): 467-71.
36. Todd, C. S., A. Nasir, M. R. Stanekzai, K. Fiekert, H. L. Sipsma, D. Vlahov, and S. A. Strathdee. "Hepatitis C and HIV Incidence and Harm Reduction Program Use in a Conflict Setting: An Observational Cohort of Injecting Drug Users in Kabul, Afghanistan." Harm Reduct J 12 (Oct 16 2015): 22.
37. Wanigaratne, S., D. C. Cole, K. Bassil, I. Hyman, R. Moineddin, and M. L. Urquia. "Contribution of HIV to Maternal Morbidity among Refugee Women in Canada." Am J Public Health 105, no. 12 (Dec 2015): 2449-56.
38. Wanigaratne, S., M. Rashid, A. Gagnon, D. C. Cole, Y. Shakya, R. Moineddin, J. Blake, et al. "Refugee Mothers, Migration Pathways and HIV: A Population-Based Cohort Study." AIDS Care 32, no. 1 (Jan 2020): 30-36.
39. Wollants, E., M. Schoenenberg, C. Figueroa, G. Shor-Posner, W. Klaskala, and M. K. Baum. "Risk Factors and Patterns of HIV-1 Transmission in the El Salvador Military During War Time." AIDS 9, no. 11 (Nov 1995): 1291-2.
40. Zea, M. C., C. A. Reisen, F. T. Bianchi, F. A. Gonzales, F. Betancourt, M. Aguilar, and P. J. Poppen. "Armed Conflict, Homonegativity and Forced Internal Displacement: Implications for HIV among Colombian Gay, Bisexual and Transgender Individuals." Cult Health Sex 15, no. 7 (2013): 788-803.
41. Zihindula, G., and P. Maharaj. "Risk of Sexual Violence: Perspectives and Experiences of Women in a Hospital in the Democratic Republic of Congo." J Community Health 40, no. 4 (Aug 2015): 736-43.

**Supplementary Appendix (Table S1). Ethical principles and practices identified in each of the included studies addressing factors associated with HIV acquisition in humanitarian crises (n=41)**


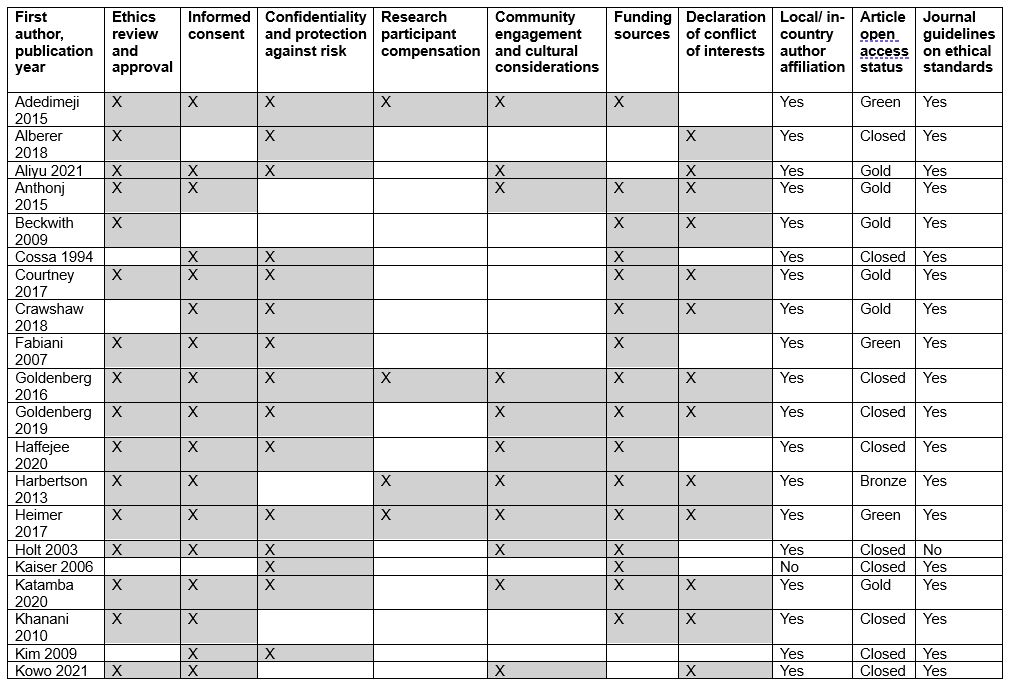


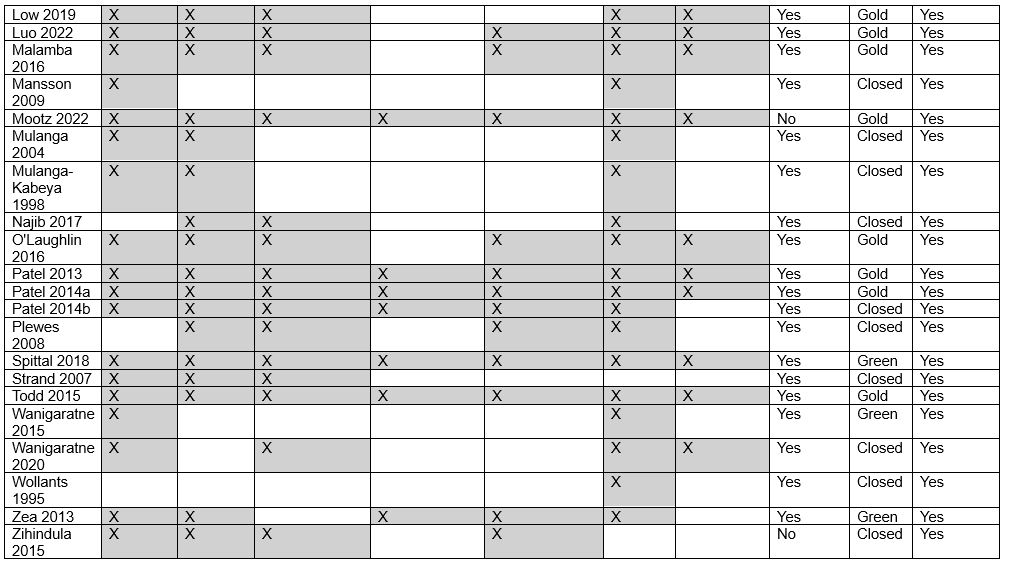


Note: “X” and grey shaded cell indicate ethical principles and practices reported in the article. The blank cell indicates the absence of information in the article that does not necessarily equate to the absence of ethical principles and practices from the study. This classification does not apply to the last 3 columns of the table (i.e., local/in-country author affiliation, article open access status, and journal guidelines on ethical standards) of which the categories were reported as observed.
